# A Public Health Approach to Antimicrobial Stewardship in Long-term Care Facilities: A Multifaceted Program in Massachusetts

**DOI:** 10.1093/cid/ciag092

**Published:** 2026-02-13

**Authors:** Kap Sum Foong, Leslie Fowle, Amanda Slider, Maureen Campion, Jessica Leaf, Melissa Cumming, Barbara Bolstorff, Christina Brandeburg, Eileen McHale, Catherine Reilly, Kirthana Beaulac, Gabriela Andujar Vazquez, Benjamin Koethe, Shira Doron, Majd Alsoubani

**Affiliations:** Division of Geographic Medicine and Infectious Diseases, Department of Medicine, Tufts Medical Center, Boston, Massachusetts, USA; Massachusetts Department of Public Health, Boston, Massachusetts, USA; Massachusetts Department of Public Health, Boston, Massachusetts, USA; Division of Geographic Medicine and Infectious Diseases, Department of Medicine, Tufts Medical Center, Boston, Massachusetts, USA; Massachusetts Department of Public Health, Boston, Massachusetts, USA; Massachusetts Department of Public Health, Boston, Massachusetts, USA; Massachusetts Department of Public Health, Boston, Massachusetts, USA; Massachusetts Department of Public Health, Boston, Massachusetts, USA; Massachusetts Department of Public Health, Boston, Massachusetts, USA; Massachusetts Department of Public Health, Boston, Massachusetts, USA; Emerson Hospital, Concord, Massachusetts, USA; Section of Infectious Disease and International Health, Dartmouth Hitchcock Medical Center, Lebanon, New Hampshire, USA; Tufts Clinical and Translational Science Institute, Tufts University, Boston, Massachusetts, USA; Division of Geographic Medicine and Infectious Diseases, Department of Medicine, Tufts Medical Center, Boston, Massachusetts, USA; Division of Geographic Medicine and Infectious Diseases, Department of Medicine, Tufts Medical Center, Boston, Massachusetts, USA

**Keywords:** long-term care, nursing home, antimicrobial stewardship, public health, antibiotic start

## Abstract

**Background:**

Benchmarking is an effective strategy to improve antibiotic use but remains underutilized in long-term care (LTC). In 2018, the Massachusetts Department of Public Health, in partnership with Tufts Medical Center, launched the Antibiotic Start (AS) Reporting Program, a statewide initiative to monitor and improve antibiotic prescribing in LTC facilities through monthly reporting and feedback, alongside other stewardship interventions.

**Methods:**

Long-term care facilities voluntarily submitted monthly AS data through an online platform. AS rates were calculated as AS per 1000 resident-days. Quarterly benchmarking reports and educational activities (introduced in 2022), including LTC office hours webinars, were conducted to support interpretation and engagement. We conducted an interrupted time-series (ITS) analysis to assess changes in beta-lactam and fluoroquinolone prescribing from 2018 to 2024 and a stratified analysis comparing AS rates between facilities with and without honor roll recognition.

**Results:**

A total of 217 LTC facilities submitted ≥1 month of AS data. The overall AS rate increased by 7%, from 7.22 to 7.70 per 1000 resident-days. Fluoroquinolone starts decreased by 36%, while beta-lactam starts increased by 26%. Interrupted time series analysis showed a significant rise in beta-lactam use following the October 2022 expansion of benchmarking reports and LTC office hours (*P* < .001). A subgroup analysis showed higher total and beta-lactam AS rates among honor roll facilities compared with nonrecognized facilities.

**Conclusions:**

The Massachusetts AS Reporting Program demonstrates that sustained, data-driven public health–academic collaboration can advance antimicrobial stewardship in LTC. By combining benchmarking, feedback, education, and recognition, public health agencies can support stewardship engagement and promote sustainable improvement in antibiotic use across resource-limited settings.

## Teaser

The Massachusetts Antibiotic Start Reporting Program demonstrates antimicrobial stewardship in long-term care is feasible through public health–academic collaboration. Benchmarking, feedback, education, and recognition were associated with improved prescribing patterns: 36% decline in fluoroquinolone use and 26% increase in beta-lactams.

Antibiotics are among the most commonly prescribed drug classes in the long-term care (LTC) setting [1]. At least half of LTC residents receive 1 or more courses of antibiotics annually, with up to three-quarters deemed unnecessary [2, 3]. Such inappropriate antibiotic use contributes to the growing crisis of antimicrobial resistance [4]. These facilities often serve as important reservoirs for multidrug-resistant organisms [5, 6]. In response, the Centers for Disease Control and Prevention (CDC) released the Core Elements of Antibiotic Stewardship for Nursing Homes in 2015 [7]. Within the Core Elements' tracking domain, monitoring antibiotic starts (ASs), defined as the initiation of an antibiotic course for an LTC resident, is recommended as a measure of antibiotic use to assess prescribing patterns and guide improvement efforts [7]. Because AS requires relatively limited effort to track and is commonly monitored through routine infection prevention surveillance in nursing homes, it represents a practical early stewardship metric [8]. The adoption of the Core Elements has gradually increased over time, likely driven in part by regulatory mandates, such as the Centers for Medicare & Medicaid Services (CMS) reform requirements [9–11]. However, the tracking and education domains remain the least adopted elements of stewardship programs in the LTC setting [9–11].

Recognizing persistent implementation gaps, national experts have called for strengthened stewardship infrastructure in LTC [4]. Multifaceted programs, such as webinars, decision aids, and expert office hours, have led to measurable improvements in prescribing [12–16]. Reported outcomes include early discontinuation of antibiotics, reduced fluoroquinolone use, and fewer unnecessary urine cultures [13–16]. However, scalability remains limited as many facilities lack infectious disease expertise, diagnostic support, and staff capacity to implement and sustain programs [17–20].

Partnerships with public health agencies have emerged as a promising strategy to address these barriers [20]. Collaborations between LTC facilities, academic institutions, and state or local health departments can provide external infrastructure, technical assistance, and analytic capacity for support of stewardship efforts [19, 21, 22]. One such approach is benchmarking, a data-driven method that allows facilities to compare prescribing practices, identify opportunities for improvement, and guide public health agencies in targeting high-priority facilities or prescribing patterns [18, 23]. Although well established in hospital settings, benchmarking remains underutilized in LTC despite its potential to support both facility- and system-level improvements.

Leveraging academic–public health collaboration, the Massachusetts Department of Public Health (DPH) launched a statewide AS benchmarking initiative alongside additional antimicrobial stewardship interventions, including educational outreach, quarterly benchmarking reports, and structured office hours. These efforts aimed to promote data transparency, deliver tailored feedback, and support stewardship engagement and practices in LTC. In this study, we describe the design and implementation of these interventions and evaluate their impact on AS trends across participating facilities from 2018 to 2024.

## METHODS

### Program Infrastructure and Overview

This program was established through a collaboration between the Massachusetts DPH Healthcare Associated Infections/Antimicrobial Resistance (HAI/AR) Team and the Antimicrobial Stewardship Team (AST) at Tufts Medical Center. Tufts Medical Center is a 415-bed academic medical center in Boston, Massachusetts. A CDC-funded contract since 2016 has supported dedicated time for 3 infectious disease physicians and a clinical pharmacist to collaborate on these stewardship efforts. In this collaboration, the Tufts AST offered subject-matter expertise; led the development of the study design, data collection, and analytic plan; and collaborated with the DPH team to create educational content and implementation supports tailored for LTC facilities.

The Massachusetts DPH HAI/AR Team maintains routine communication with all licensed LTC facilities in the state through established public health listservs and regulatory communication channels, which were leveraged for statewide outreach. As such, all Massachusetts LTC facilities were eligible and invited to participate in the AS Reporting Program.

Relationships with LTC facilities were cultivated iteratively through repeated program touchpoints, including statewide outreach, benchmarking feedback, educational activities, and engagement via the Infection Control Assessment and Response (ICAR) program, rather than through a single enrollment effort.

### Antibiotic Start Reporting Program

The AS Reporting Program was launched in 2018 to create a standardized mechanism for tracking antibiotic initiation in LTC. Facilities voluntarily submitted monthly data through an online platform (initially SurveyMonkey, later ArcGIS Survey 123) [24]. Submitted data included facility characteristics, submitter demographics, local LTC consultant pharmacist's contact information, reporting month, bed size, resident census (short vs long stay), service types (eg, long-term care, ventilator, and hospice/palliative), monthly resident-days, and AS by class. Antibiotic start data were entered manually by facility-designated staff using existing infection prevention surveillance data or pharmacy records; the program did not require extraction from electronic health records and was designed to accommodate facilities with varying levels of health information technology capacity. Antibiotic start rates were calculated by dividing the total number of AS by the number of resident-days for the reporting period and were normalized as AS per 1000 resident-days. Facilities could report data for any month within a quarter, and data entry remained open to accommodate reporting delays and ensure inclusion in longitudinal analyses.

### Antimicrobial Stewardship Interventions

Following the initial program rollout, Massachusetts DPH and the Tufts AST implemented a series of phased, multifaceted interventions designed to promote reporting participation and improve stewardship practices across LTC facilities. Early phases focused primarily on establishing reporting infrastructure and re-engaging facilities, while later phases emphasized benchmarking feedback, education, and targeted prescribing guidance. Table 1 summarizes the timing, components, and scope of these interventions.

**Table 1.** Massachusetts Department of Public Health-Led Antimicrobial Stewardship Interventions Implemented Over Time in Long-term Care Facilities, 2018–2024

Intervention	Implementation Timeframe	Primary Program Components	Scope
Program initiation and reporting infrastructure	2018	Voluntary Antibiotic Start Reporting Program launched; standardized monthly antibiotic start data submission via online platform; baseline outreach and technical support	Reporting-focused
Participation re-engagement and enrollment support	January 2019	Targeted outreach via stewardship listserv, email, and phone; on-site Infection Control Assessment and Response visits by DPH staff to encourage participation	Participation-focused
Benchmarking feedback and recognition	January 2022	Distribution of facility-level benchmarking reports; introduction of LTC honor roll (gold/silver/bronze) to incentivize sustained reporting	Feedback and incentive-based motivation
Expanded stewardship education and prescribing support	October 2022	Dissemination of stewardship toolkits (eg, beta-lactam allergy evaluation and high utilization guidance); 9-month webinar series evolving into monthly LTC office hours; enhanced benchmarking reports with longitudinal trend displays	Multifaceted stewardship intervention

Abbreviations: DPH, Department of Public Health; LTC, long-term care.

In January 2019, Massachusetts DPH initiated a targeted re-engagement effort after observing a decline in facility participation in the program. Strategies included periodic outreach through a stewardship listserv, direct follow-up via phone and email, and on-site ICAR visits (limited to facilities identified by public health need) by DPH epidemiologists and public health nurses, to encourage enrollment in the AS Reporting Program. During this initial period, program efforts were limited to supporting data submission and did not include formal prescribing feedback, educational interventions, or incentives beyond access to the reporting platform.

In January 2022, Massachusetts DPH and the Tufts AST introduced and distributed benchmarking reports (initially monthly and then quarterly) to participating facilities. Reports provided individualized feedback comparing each facility's AS rate to the statewide aggregate for that quarter. Facilities that submitted at least 1 month of data received these reports and were encouraged through reminder communications to sustain reporting. An example benchmarking report is provided in Supplementary Figure 1. To further incentivize engagement, an LTC honor roll program was introduced, annually recognizing facilities based on reporting frequency within a calendar year: gold for submission in all 12 months, silver for ≥9 months, and bronze for ≥6 months. Honorees were acknowledged publicly on a dedicated Massachusetts DPH website and received certificates recognizing their commitment to antimicrobial stewardship.

In October 2022, the program was expanded to include more intensive stewardship support. Massachusetts DPH and the Tufts AST collaboratively developed and disseminated stewardship resources, including a beta-lactam allergy evaluation toolkit, a comparison guide to better understand treatment versus surveillance criteria, infection management guidelines, and prescribing recommendations, such as encouraging the use of shorter, effective antibiotic courses for common infections. Particular emphasis was placed on strategies to reduce high-use agents, such as fluoroquinolones, in favor of beta-lactam antibiotics where appropriate. This included a high utilization guidance document outlining risks of fluoroquinolone use and specific actions to reduce their use [25, 26].

These materials were hosted on a dedicated Massachusetts DPH website [27]. A 9-month educational webinar series was delivered, covering foundational to advanced topics in antimicrobial stewardship and infection prevention, and supplemented by reminders to submit AS data. This series later evolved into recurring monthly LTC office hours webinars, featuring brief didactic presentations, open Q&A, case discussions, and peer sharing, with content focused on diagnosis and treatment of common infections and infection prevention topics relevant to LTC. A complete list of topics covered during LTC office hours is provided in Supplementary Table 1. Sessions were intended for LTC providers, including nurses, infection preventionists, pharmacists, prescribing clinicians, and administrators, with an average of 60–80 facilities participating per session. Outreach was conducted broadly through statewide listserv communications, with participation open to all facilities regardless of AS reporting status. Concurrently, benchmarking reports were refined to display quarterly prescribing trends over the prior year (Supplementary Figure 2). Reports included facility-specific and statewide AS rates, with longitudinal graphs highlighting overall use and rates for key antibiotic classes, notably fluoroquinolones and third-generation cephalosporins.

### Study Design

We conducted a pre–postintervention study using voluntarily submitted data from January 2018 to December 2024. All LTC facilities in Massachusetts (∼400) were eligible to report data. We excluded duplicate monthly entries, records with missing resident-days, and presumed erroneous data entry, such as resident-days < 250 or >10 000 per month and entries with >100 AS for a single agent. In total, 53 entries were excluded based on these criteria. Facilities could report retrospectively and were not required to submit data consecutively.

### Outcomes and Definitions

The primary outcome was facility-level monthly AS rates. Antibiotic classes analyzed included penicillin/beta-lactamase inhibitors, first- through third-generation cephalosporins, fluoroquinolones, sulfonamides, nitrofurantoin, macrolides, tetracyclines, clindamycin, and glycopeptides. The reporting tool captured both oral and intravenous antibiotics; however, fourth-generation cephalosporins and carbapenems were rarely reported in this setting and therefore were not analyzed as separate classes. For the purpose of this study, beta-lactams were defined as penicillins and first- through third-generation cephalosporins. Intervention time points were January 2019 (re-engagement initiatives), January 2022 (quarterly benchmarking reports and reminders), and October 2022 (educational outreach and LTC office hours webinars).

### Statistical Analysis

Descriptive statistics were used to summarize facility participation and antibiotic use. We performed interrupted time-series (ITS) analyses using segmented linear regression to evaluate changes in monthly fluoroquinolone and beta-lactam AS rates. Models included a continuous time variable to estimate baseline preintervention trends and indicator variables representing each intervention. For each intervention, models estimated both an immediate-level change (step change) and a change in slope (trend) following the intervention. Postintervention slopes were calculated as the sum of the baseline slope and corresponding postintervention coefficients. Beta-lactam and fluoroquinolone AS rates were modeled separately.

We also performed a subgroup analysis comparing median AS rates (overall and by class) between facilities achieving honor roll recognition (gold, silver, or bronze) and those without recognition using the Wilcoxon rank-sum test. This subgroup analysis explored whether greater engagement with the DPH stewardship program, reflected by consistent reporting, was associated with differences in prescribing patterns, measured by total, beta-lactam, and fluoroquinolone AS rates. Honor roll tiers were not analyzed separately, as they reflect reporting frequency rather than distinct stewardship interventions. All analyses were conducted using SAS version 9.4 (SAS Institute Inc., Cary, NC). All *P* values were from 2-sided tests, with results deemed statistically significant at *P* ≤ .05. This study was reviewed and deemed exempt by the Massachusetts DPH and Tufts Medical Center Institutional Review Board.

## RESULTS

**Figure 1.**
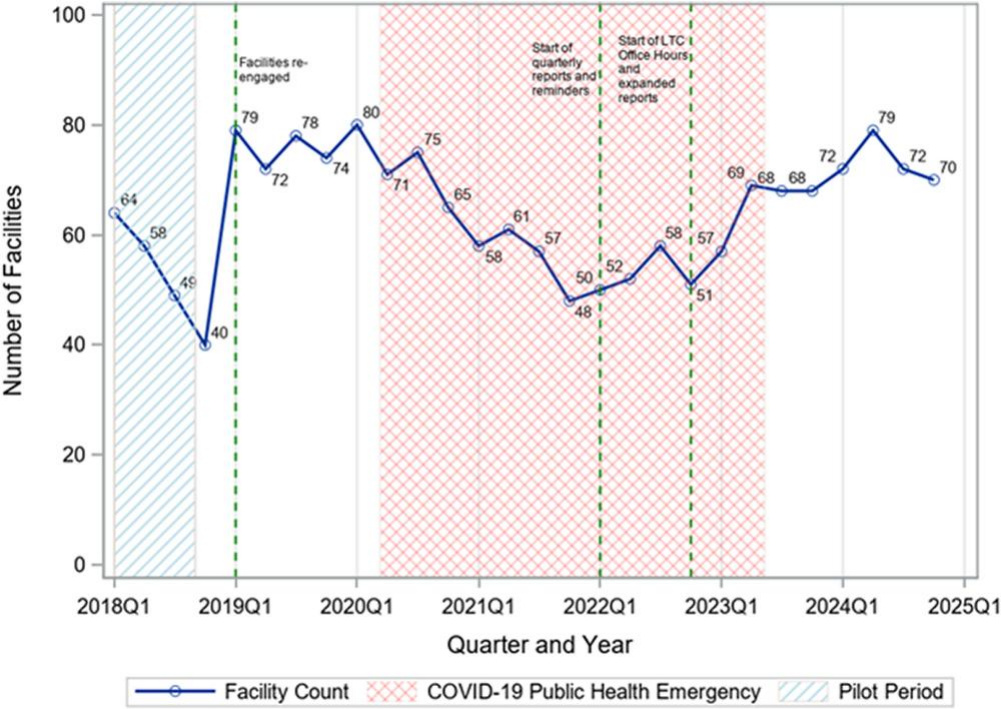
Number of long-term care facilities reporting by quarter and year, 2018–2024. Abbreviations: COVID-19, coronavirus disease 2019; LTC, long-term care.

From 2018 to 2024, a total of 217 Massachusetts LTC facilities submitted at least 1 month of AS data, representing 12 518 102 resident-days. The median number of reporting facilities per month was 53 (interquartile range [IQR], 46–65), and participation increased from 18.8% to 24.2% of all LTC facilities statewide (Figure 1). Among participating facilities, the median bed size was 118 (IQR, 81–137); 96.1% provided long-term general nursing care, 75.4% reported hospice or palliative care services, and 2.2% offered ventilator services (Table 2). Short-stay residents comprised 22.4% of the total census among facilities reporting both short- and long-term populations each month.

**Table 2.** Characteristics of Participating Long-term Care Facilities in Massachusetts, 2018–2024

Characteristic	Number of Facilities (n = 217)
Certified beds in facility, median (IQR)	118 (81–137)
Services provided**^a^**, n (%)	
Long-term general nursing care	172 (96.1)
Skilled nursing/short-term (subacute) rehabilitation	169 (94.4)
Hospice/palliative care	135 (75.4)
Long-term dementia	124 (69.3)
Bariatric	33 (18.4)
Long-term psychiatric (nondementia)	20 (11.2)
Ventilator	4 (2.2)
Honor roll status, n (%)	
Gold	58 (26.7)
Silver	14 (6.5)
Bronze	14 (6.5)
No honor roll	131 (60.4)

^a^The number of facilities reporting services was 179. Percentages were calculated among facilities reporting data for each characteristic. Missing data were excluded from the denominators.

**Figure 2.**
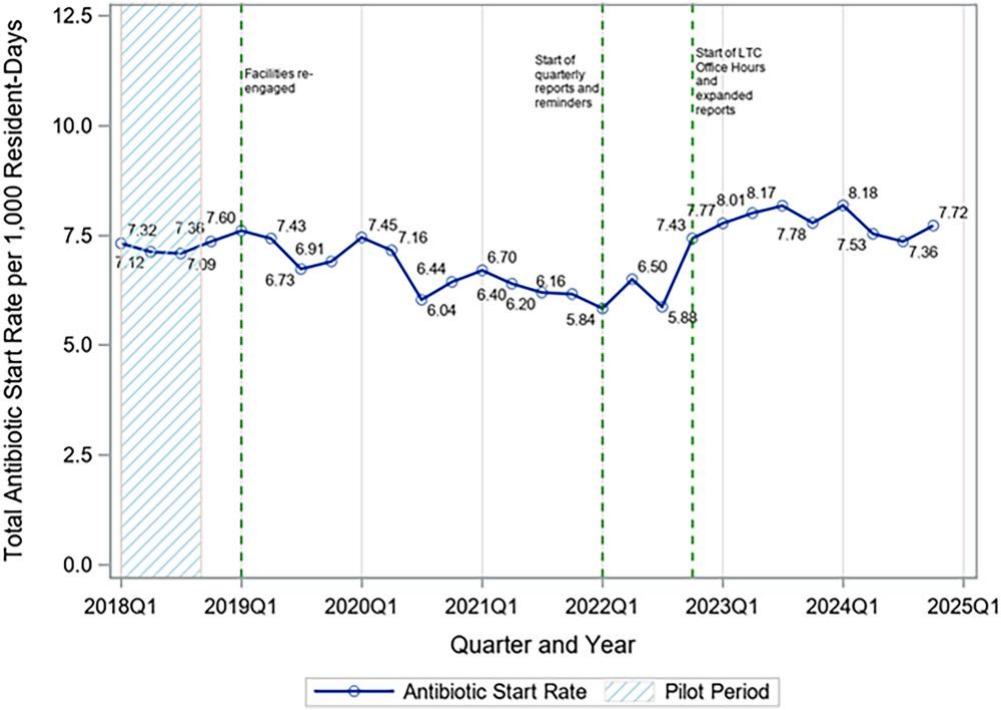
Total antibiotic start rate per 1000 resident-days by quarter and year, 2018–2024. Abbreviation: LTC, long-term care.

**Figure 3.**
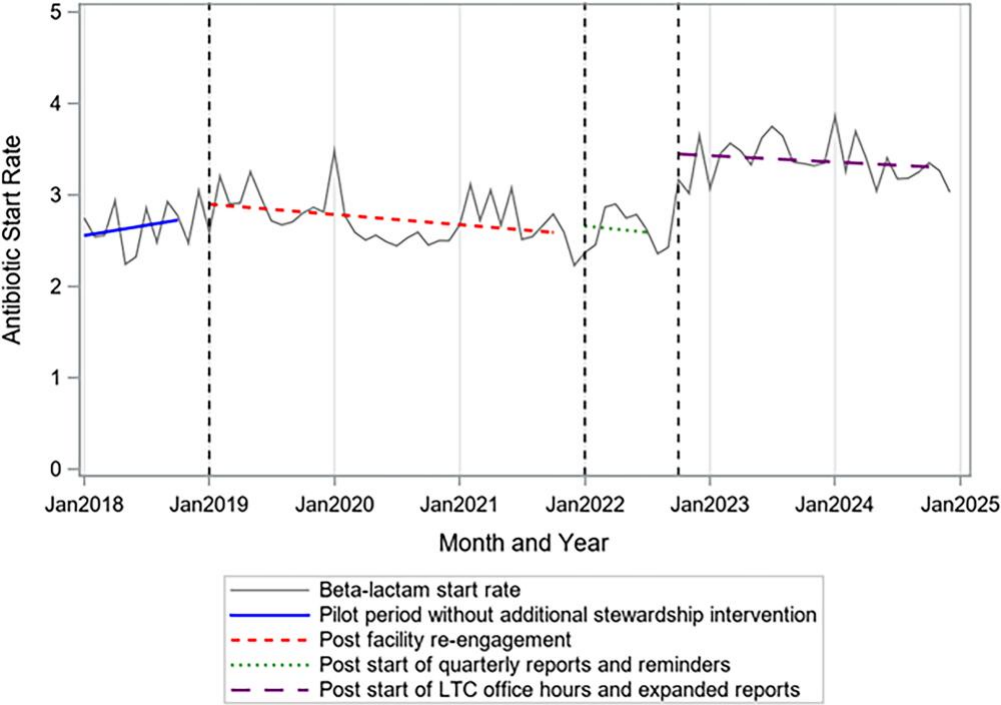
Beta-lactam start rates with intervention segments by month, 2018–2024. Abbreviation: LTC, long-term care.

**Figure 4.**
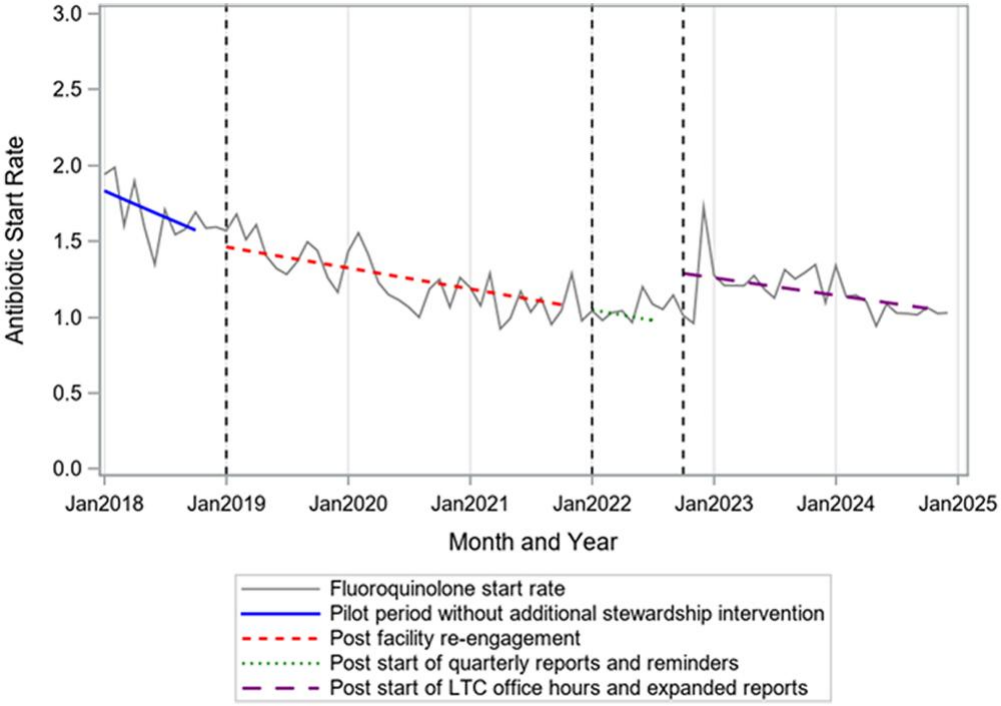
Fluoroquinolone start rates with intervention segments by month, 2018–2024. Abbreviation: LTC, long-term care.

Over the study period, the overall AS rate increased by 7%, from 7.22 to 7.70 starts per 1000 resident-days (Figure 2). The most frequently prescribed antibiotic classes were penicillin/beta-lactamase inhibitors, first- and third-generation cephalosporins, fluoroquinolones, and tetracycline, which together accounted for 62.7% of all AS. Fluoroquinolone use declined by 36% from 1.68 to 1.08 starts per 1000 resident-days, while beta-lactam use increased by 26%, from 2.64 to 3.33 starts per 1000 resident-days (Figures 3 and 4).

Interrupted time-series analysis showed an immediate significant increase (step change) in beta-lactam start rates following the October 2022 implementation of LTC office hours webinars and the expanded benchmarking reports (*P* < .001). No specific intervention was significantly associated with the observed decline in fluoroquinolone prescribing. Segmented regression plots illustrating level and slope changes for beta-lactam and fluoroquinolone AS rates are shown in Figures 3 and 4, with corresponding parameter estimates presented in Supplementary Tables 2 and 3.

In a subgroup analysis, among the 217 participating facilities, 86 (39.6%) achieved honor roll recognition based on reporting frequency: 58 (26.7%) achieved gold-tier status, 14 (6.5%) silver, and 14 (6.5%) bronze. Honor roll facilities had a median total AS rate of 6.5 (IQR, 4.1–8.8) per 1000 resident-days compared with 5.7 (IQR, 3.7–8.1) among nonhonoree facilities (*P* = .005). Median beta-lactam AS rates were 2.8 (IQR, 1.8–3.7) versus 2.3 (IQR, 1.4–3.3) per 1000 resident-days (*P* < .001), and median fluoroquinolone AS rates were 1.0 (IQR, 0.6–1.7) versus 0.9 (IQR, 0.5–1.5) per 1000 resident-days (*P* = .032) for honoree and nonhonoree facilities, respectively.

## DISCUSSION

This statewide benchmarking initiative offers important insights into how antimicrobial stewardship can be operationalized across LTC facilities through sustained public health–academic collaboration and multiple, complementary intervention strategies. Beyond numerical trends, its success highlights the value of embedding stewardship within a continuous and data-driven feedback loop that integrates reporting, benchmarking, education, and recognition. Prior LTC stewardship interventions often relied on 1-time educational sessions or bundled strategies without mechanisms for sustainability and therefore could not be readily replicated in routine practice [12–16, 28]. In contrast, the Massachusetts program has been sustained for over 6 years by embedding stewardship activities within the state public health infrastructure. Unlike earlier national efforts, such as the Agency for Healthcare Research and Quality (AHRQ) Safety Program, which delivered a successful but time-limited educational intervention with benchmarking and implementation support [23]. That initiative operated as a centrally coordinated project without ongoing alignment with local health systems or public health priorities. The Massachusetts experience demonstrates how stewardship activities can be sustained through continued, iterative engagement.

In this program, facility-submitted AS data were transformed into individualized benchmarking reports and supported by real-time public health engagement through educational webinars and recurring LTC office hours, which functioned as peer-learning forums. These components were implemented sequentially and expanded over time, with overlap across program periods, precluding attribution of observed changes to any single intervention. Rather, the program reflects a longitudinal, evolving approach in which layered strategies collectively supported stewardship engagement and practice change.

Although the overall AS rate increased over the study period, this finding should be interpreted cautiously and in context. Reporting was voluntary and incomplete, and participation varied over time, raising the possibility that observed changes in AS rates reflect shifts in the composition of participating facilities rather than direct effects of specific interventions. Increases in reporting during later program periods coincided with expanded educational activities and LTC office hours. Despite these limitations, we observed meaningful changes in prescribing patterns, including a substantial decline in fluoroquinolone use and a concurrent increase in beta-lactam prescribing. This pattern is consistent with the intended stewardship outcomes reflecting safer, guideline-concordant prescribing practices. The increase in beta-lactam use may also reflect improved provider confidence, supported by program resources that encouraged appropriate antibiotic selection and discouraged reliance on high-risk agents, such as fluoroquinolones, which are associated with clinically significant adverse effects. Similar class-level trends have been reported in other LTC stewardship studies [12, 28].

The program's ability to promote engagement outside of formal regulatory enforcement is notable. Although antimicrobial stewardship requirements for nursing homes have been mandated by CMS since 2017 and are assessed through state survey processes, the AS Reporting Program operated separately from regulatory inspection and enforcement activities [29]. Participation was voluntary and not tied to licensure, reimbursement, or survey outcomes. Within this context, the honor roll system provided a visible, low-cost recognition framework that leveraged peer comparison rather than penalties. This aligns with findings from the AHRQ Safety Program for improving antibiotic use, in which LTC facilities were more motivated by shared learning and recognition than top-down mandates [28]. Reporting participation temporarily declined during the Coronavirus disease 19 public health emergency, consistent with CDC reports of stewardship program disruptions in LTC settings [30]. However, the program infrastructure and stewardship activities remained active, and participation rebounded as facilities recovered operational capacity, highlighting the importance of LTC-specific public health infrastructure in supporting stewardship continuity and recovery.

Facilities achieving honor roll recognition reported higher AS rates, which may reflect more complete and consistent reporting over time. Honor roll facilities may therefore represent a more engaged subset of participants, and differences in prescribing patterns between honoree and nonhonoree facilities may reflect differential responsiveness to stewardship activities rather than uniform effects across all facilities. As such, improvements observed at the program level may be driven in part by highly engaged facilities. Differences were most pronounced for beta-lactam prescribing, while differences in fluoroquinolone use were small in absolute terms (increased by 0.1 per 1000 resident-days). Collectively, these class-specific and engagement-related patterns suggest that sustained participation may amplify stewardship impact, even if overall AS rates have not uniformly declined.

Despite these successes, several limitations should be acknowledged. First, the voluntary nature of participation may introduce selection bias, as facilities with stronger leadership engagement or existing stewardship culture may have been more likely to participate, potentially limiting generalizability. Second, antibiotic appropriateness was not assessed due to the absence of resident-level clinical data. Although reductions in high-risk agents are generally desirable, increases in beta-lactam use should be interpreted cautiously without diagnostic context or outcome data. This limitation has since been addressed through updates to the survey tool that capture treatment indication and days of therapy, enabling more precise stewardship evaluations in future analyses. Third, some data submissions were incomplete or inconsistently reported. Although thresholds were applied to exclude implausible entries, future efforts should incorporate automated data pipelines via electronic health records. As an ecological design, causal inference is limited. While ITS methods accounted for baseline trends, unmeasured confounders and concurrent events may have influenced results. Finally, the honor roll's influence on behavior versus its reflection of preexisting motivation warrants further exploration.

In summary, the Massachusetts Antibiotic Start Reporting Program demonstrates that antimicrobial stewardship in LTC is feasible through sustained public health–academic collaboration and a multicomponent approach. Its success derives from the combined impact of benchmarking, feedback, education, and recognition implemented over time, rather than from any single discrete intervention. Observed changes occurred in the context of overlapping strategies, and antibiotic prescribing appropriateness could not be directly assessed in this analysis. Nonetheless, this experience offers practical lessons for engaging LTC facilities at scale and underscores the critical role of public health infrastructure in supporting stewardship implementation and sustainment in resource-limited settings.

## Supplementary Material

ciag092_Supplementary_Data

## Supplementary Data

Supplementary materials are available at *Clinical Infectious Diseases* online. Consisting of data provided by the authors to benefit the reader, the posted materials are not copyedited and are the sole responsibility of the authors, so questions or comments should be addressed to the corresponding author.
