# Correction to: A Public Health Approach to Antimicrobial Stewardship in Long-term Care Facilities: A Multifaceted Program in Massachusetts

**DOI:** 10.1093/cid/ciag441

**Published:** 2026-07-22

This is a correction to: Kap Sum Foong, Leslie Fowle, Amanda Slider, Maureen Campion, Jessica Leaf, Melissa Cumming, Barbara Bolstorff, Christina Brandeburg, Eileen McHale, Catherine Reilly, Kirthana Beaulac, Gabriela Andujar Vazquez, Benjamin Koethe, Shira Doron, Majd Alsoubani, A Public Health Approach to Antimicrobial Stewardship in Long-term Care Facilities: A Multifaceted Program in Massachusetts, *Clinical Infectious Diseases*, 2026; https://doi.org/10.1093/cid/ciag092

The following change has been made to the originally published paper.

Under **Notes**, and ***Financial support.*** the following funding statement has been added: “K.S.F. was supported by the National Center for Advancing Translational Sciences, National Institutes of Health (NIH), Award Number 1K12TR004384. The content is solely the responsibility of the authors and does not necessarily represent the official views of the NIH.”

