# Supplementary material for: A Public Health Approach to Antimicrobial Stewardship in Long-term Care Facilities: A Multifaceted Program in Massachusetts
Source: Clin Infect Dis. 2026 Feb 13;83(1):e10–7. doi: 10.1093/cid/ciag092 (PMC13087996; doi:10.1093/cid/ciag092)
Supplement: ciag092_Supplementary_Data [file ciag092_supplementary_data.docx]

**Supplementary Figure 1**. Example of an antibiotic start benchmarking report provided to participating long-term care facilities, 2018 to 2021
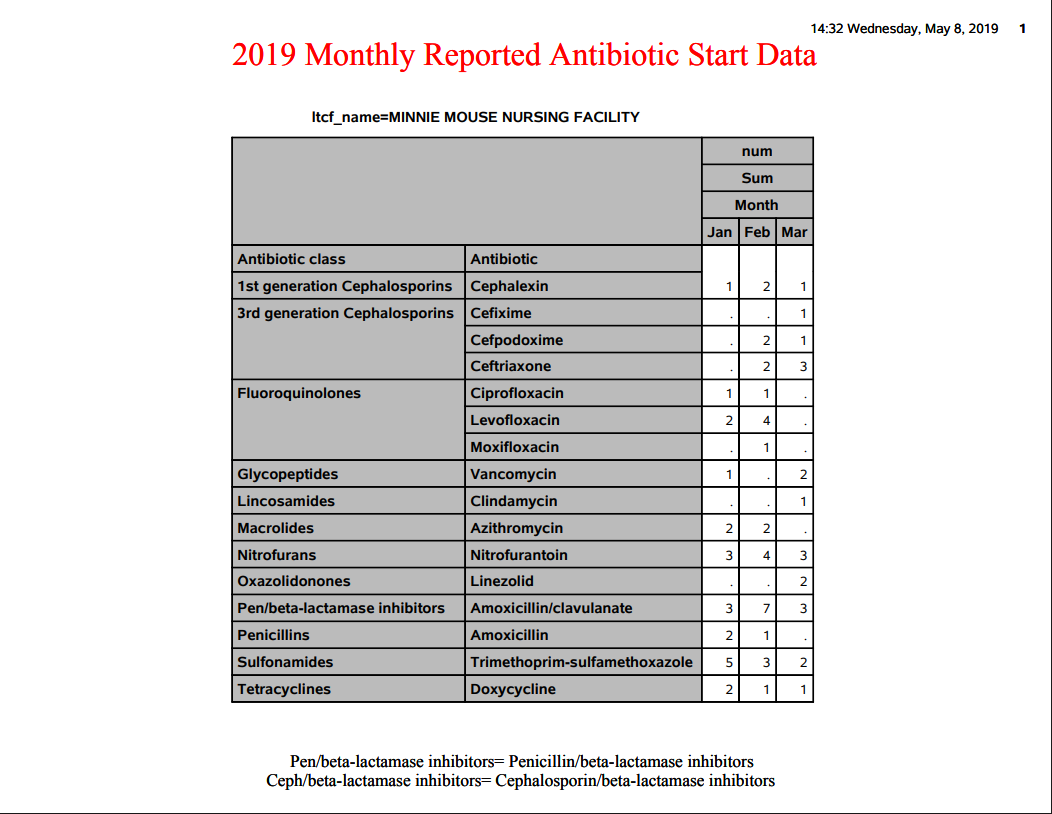

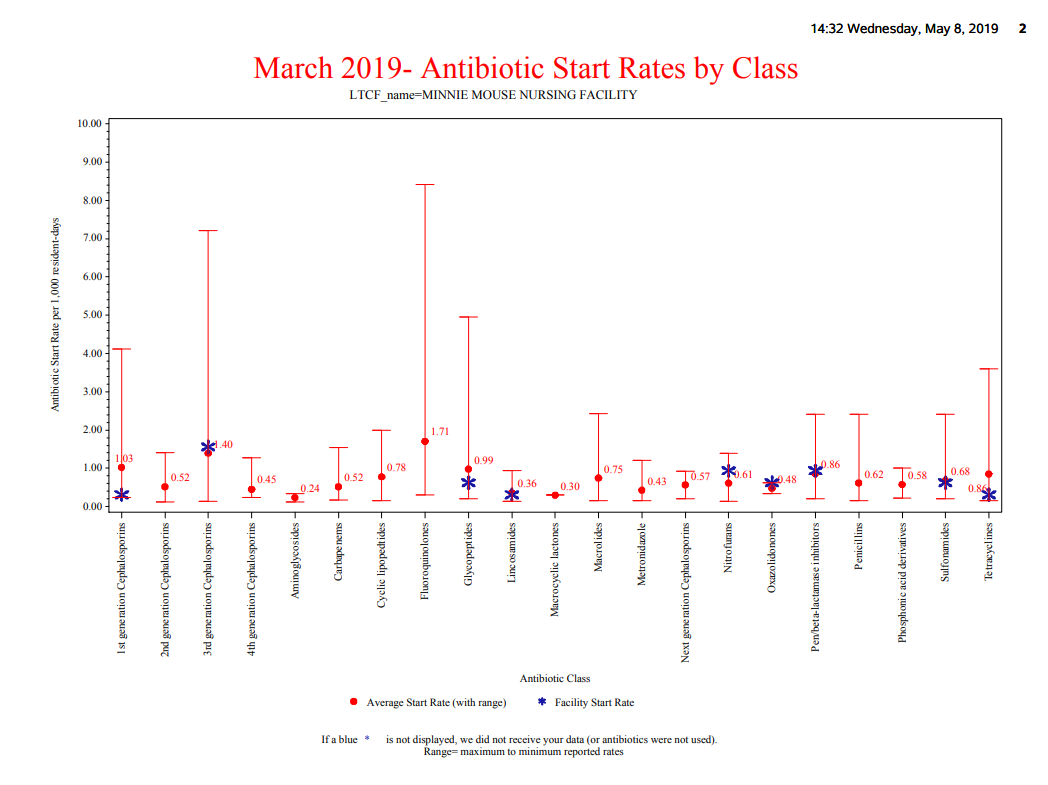


**Supplementary Figure 2.** Example of an expanded antibiotic start benchmarking report provided to participating long-term care facilities, 2022 to 2024


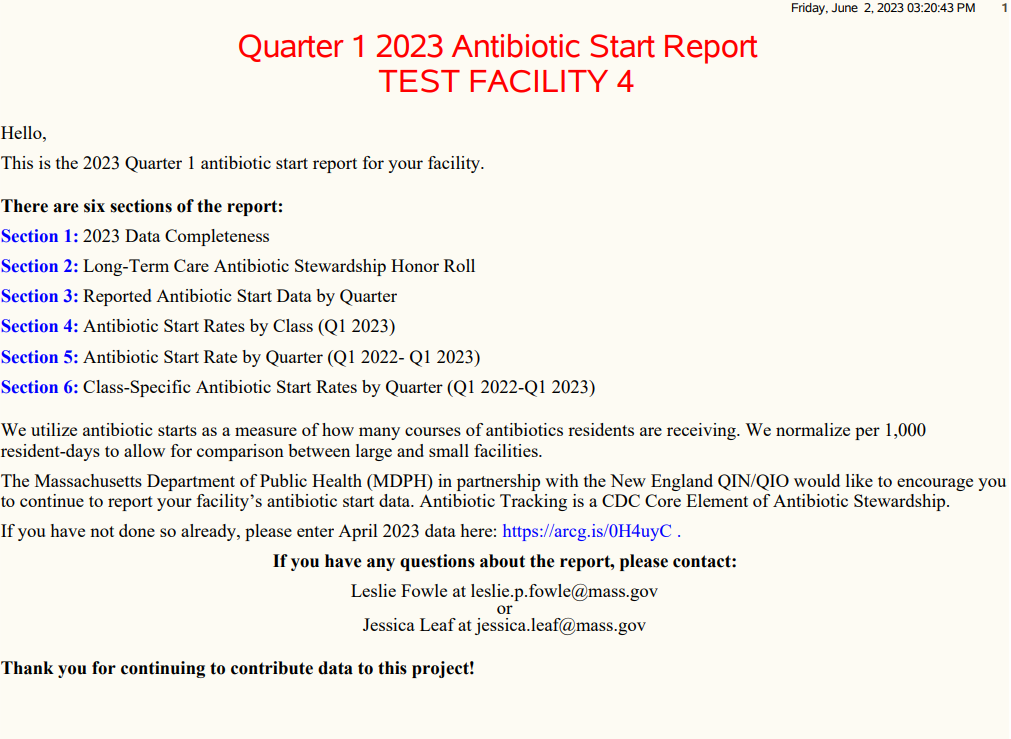


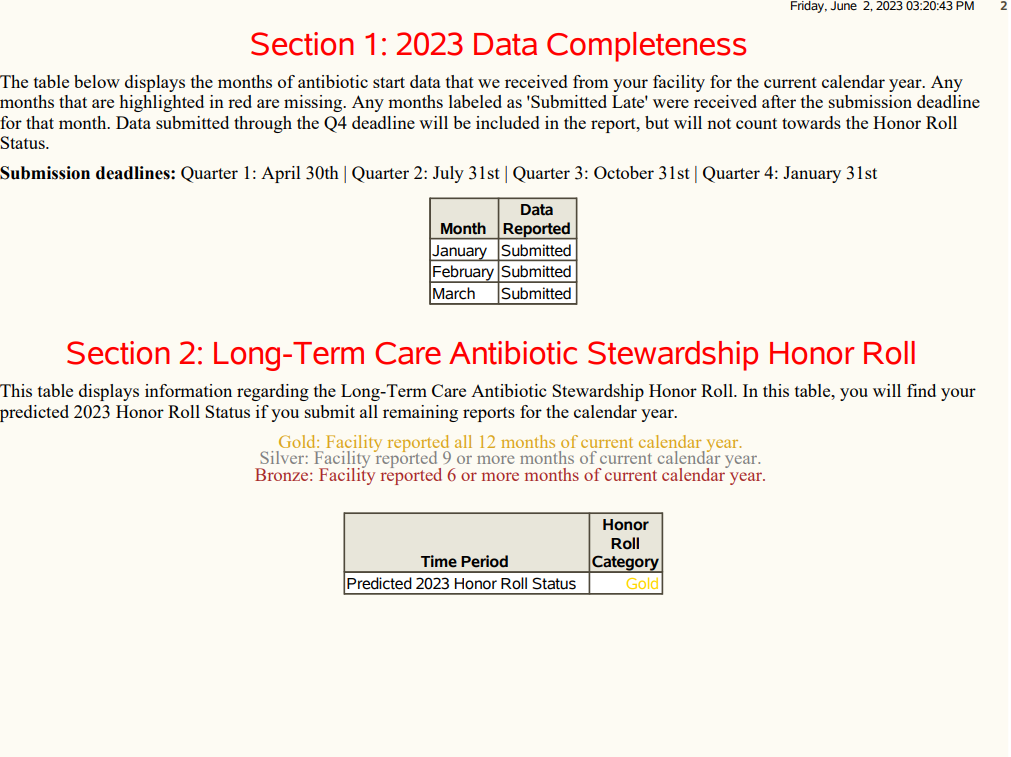


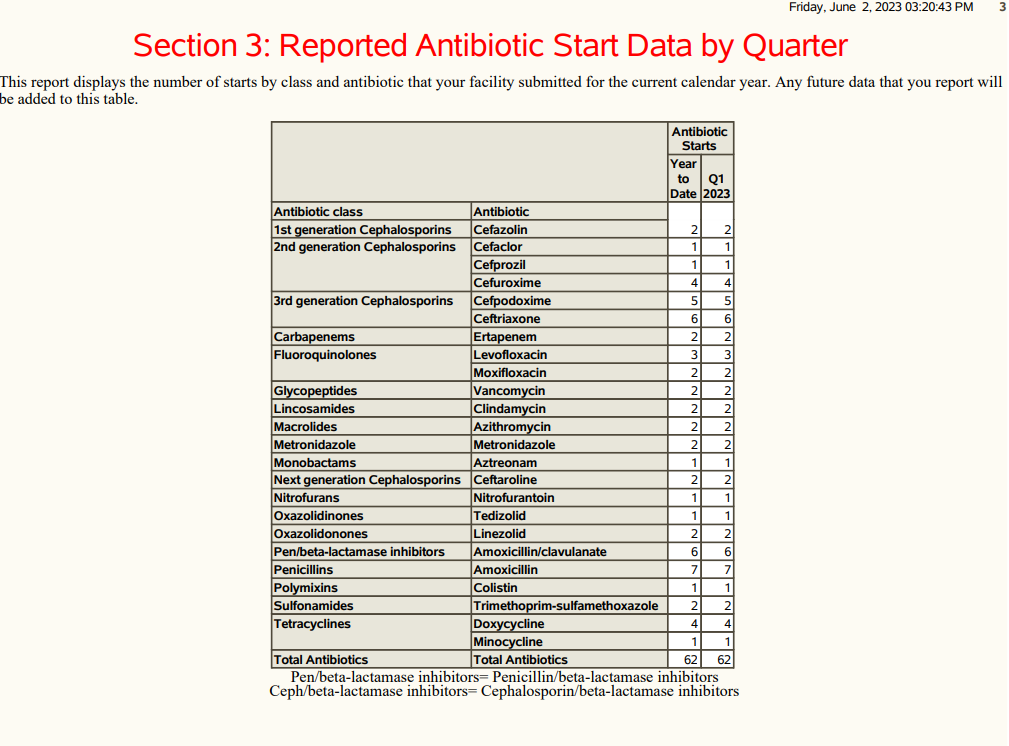


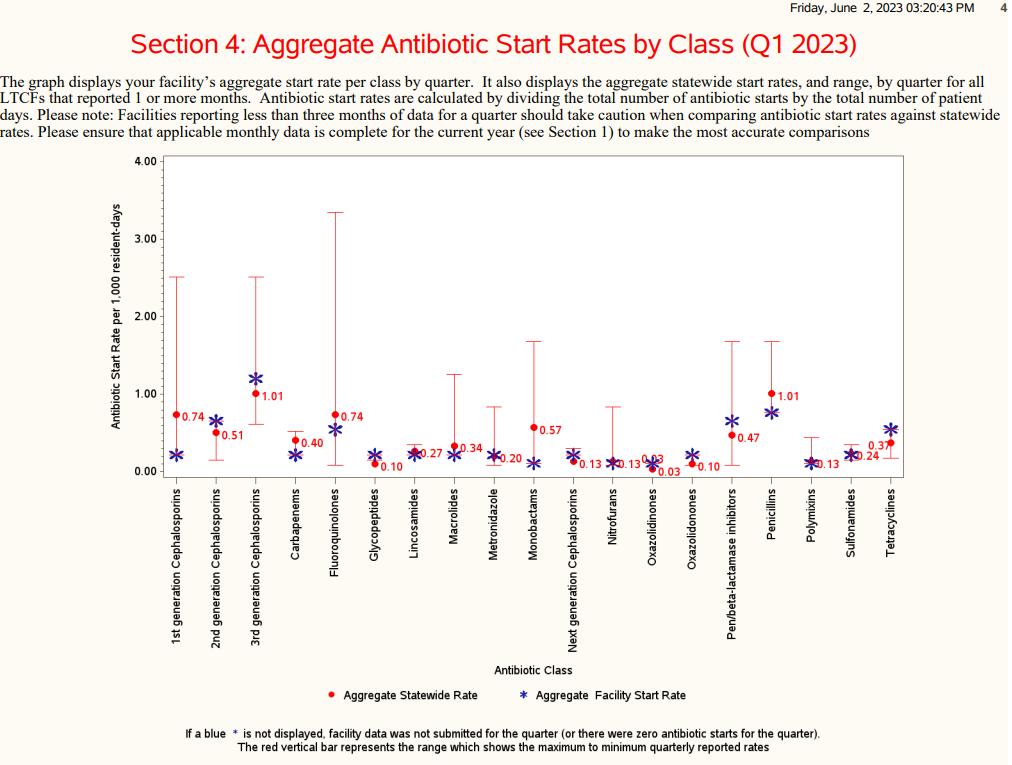


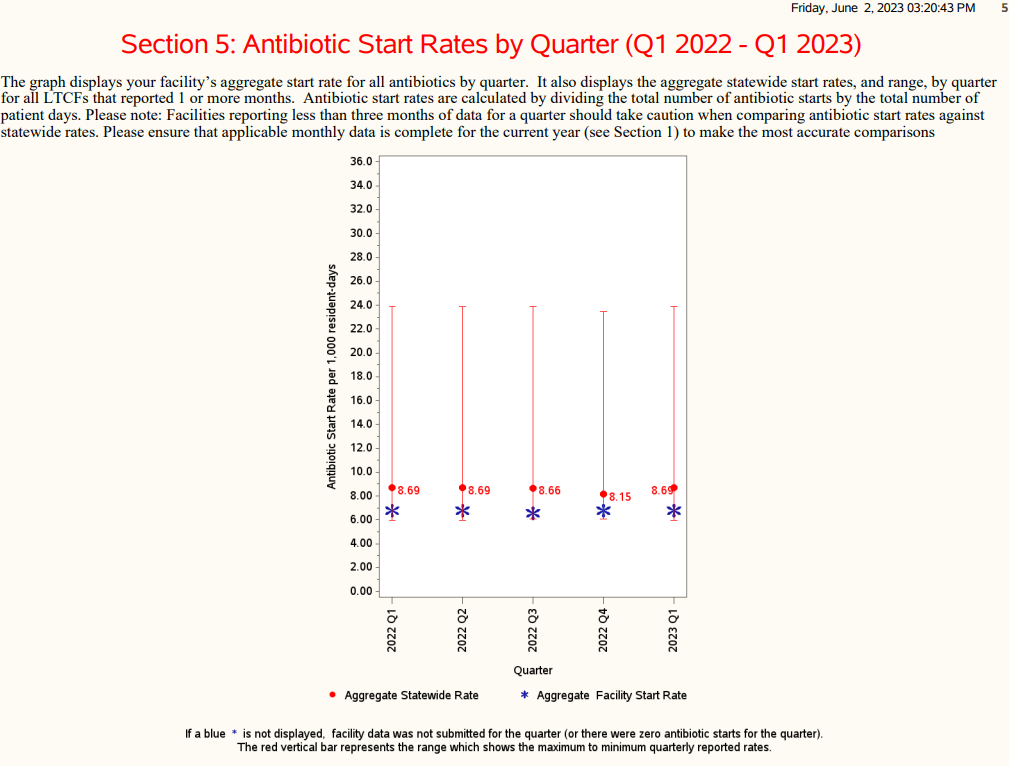


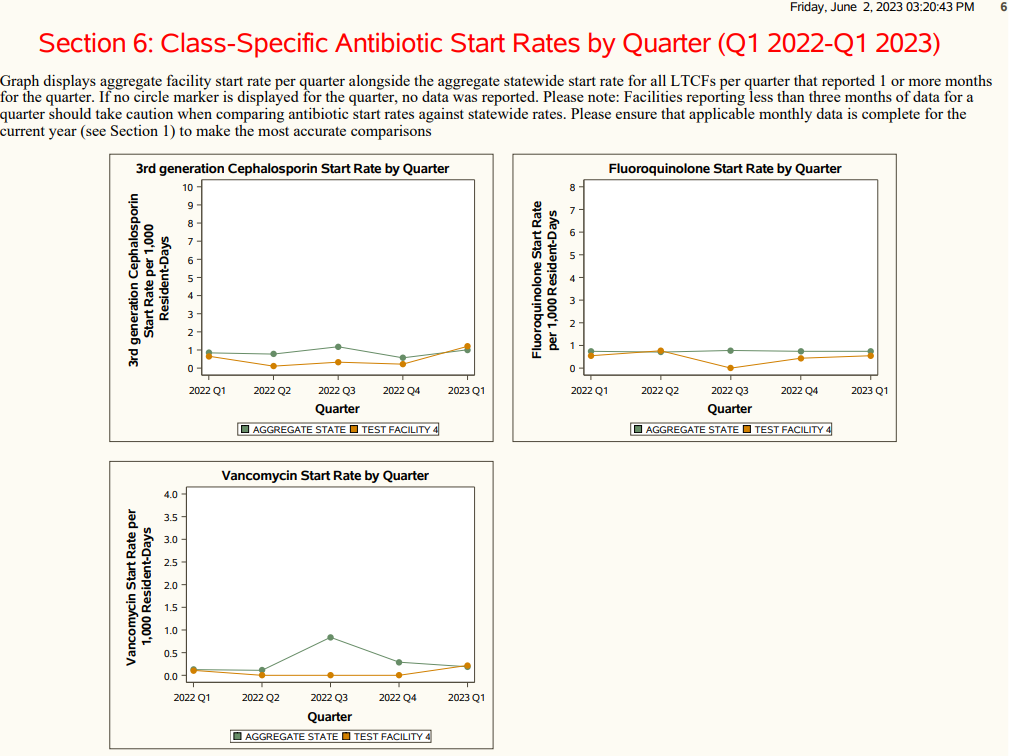


**Supplementary Table 1.** Antimicrobial stewardship and infection prevention topics covered during long-term care office hours

| - Antibiotic Use in End-of-Life Care - Antimicrobial Stewardship Opportunities and Progress: U.S. Antibiotic Awareness Week Kickoff - Asymptomatic Bacteriuria vs. UTI - *Clostridioides difficile* and Antibiotic Stewardship - De-Escalation and Duration of Antibiotic Therapy - Educating Residents and Families on Antibiotic Use - Enhanced Barrier Precautions - Germs are Everywhere: The Role of the Environment in the Transmission of MDROs - How to Interpret Your Antibiotic Start Reports - How to Request and Use an Antibiogram - How to Start an Antibiotic Stewardship Program - How to Use the Loeb vs. McGeer Criteria - Infection Prevention and Control in Skilled Nursing Facilities - Leveraging Your Data: Antibiotic Start Reporting Program and Antibiotic Allergy ​De-labeling Project - Managing A Penicillin Allergy - Overview of Invasive Group A *Streptococcus* (GAS) for Long-Term Care Facilities - Pneumonia and Antibiotic Stewardship - Post-Acute Care Transitions - Respiratory Viral Illness Update - Shorter is Smarter: Choosing the Right Duration of Therapy for Antibiotics - Skin and Soft Tissue Infection and Antibiotic Stewardship - Standard, Transmissions-Based, and Enhanced Barrier Precautions - The Role of Diagnostics in Reducing Antibiotic Use - Tracking Antibiotic Use with the Improved Antibiotic Start Reporting Program - U.S. Antibiotic Awareness Week 2023 – How to Get Involved |
| --- |

**Supplementary Table 2**. Interrupted time series segmented regression estimates of changes in beta-lactam antibiotic start rates following stewardship interventions

|  | Parameter estimate (95% CI) | *p* value |
| --- | --- | --- |
| Intercept pre-intervention | 2.539 (2.242, 2.837) | <.0001 |
| Pre-intervention segment slope | 0.019 (-0.022, 0.059) | 0.360 |
| Level change post facility re-engagement | 0.145 (-0.165, 0.456) | 0.353 |
| Change in slope post facility re-engagement | -0.028 (-0.069, 0.013) | 0.180 |
| Level change post start of quarterly reports and reminders | 0.099 (-0.286, 0.485) | 0.609 |
| Change in slope post start of quarterly reports and reminders | -0.002 (-0.065, 0.061) | 0.947 |
| Level change post start of LTC office hours and expanded antibiotic start report | 0.884 (0.530, 1.238) | <.0001 |
| Change in slope post start of LTC office hours and expanded antibiotic start report | 0.006 (-0.058, 0.069) | 0.862 |

Abbreviation: CI, confidence interval; LTC, long-term care.

**Supplementary Table 3**. Interrupted time series segmented regression estimates of changes in fluoroquinolone antibiotic start rates following stewardship interventions

|  | Parameter estimate (95% CI) | *p* value |
| --- | --- | --- |
| Intercept pre-intervention | 1.861 (1.694, 2.028) | <.0001 |
| Pre-intervention segment slope | -0.029 (-0.051, -0.006) | 0.014 |
| Level change post facility re-engagement | 0.007 (-0.166, 0.181) | 0.932 |
| Change in slope post facility re-engagement | 0.014 (-0.009, 0.037) | 0.236 |
| Level change post start of quarterly reports and reminders | 0.0003 (-0.216, 0.216) | 0.998 |
| Change in slope post start of quarterly reports and reminders | 0.030 (-0.005, 0.065) | 0.095 |
| Level change post start of LTC office hours and expanded antibiotic start report | 0.178 (-0.020, 0.376) | 0.078 |
| Change in slope post start of LTC office hours and expanded antibiotic start report | -0.025 (-0.060, 0.011) | 0.174 |

Abbreviation: CI, confidence interval; LTC, long-term care.
